# The relationship of platelet-to-lymphocyte ratio with cognitive decline in T2DM

**DOI:** 10.1186/s13098-021-00772-y

**Published:** 2021-12-24

**Authors:** Licheng Du, Xueting Hu, Beibei Zhang, Xiaqi Miao, Jianing Wang, Jiamin Shen, Keke Ding, Tian Zeng, Fangyue Sun, Hong Yang, Hai Lin

**Affiliations:** 1 Department of Endocrinology, The Third Affiliated Hospital of Wenzhou Medical University, NO.108 Wansong Road, Wenzhou, 325000 Zhejiang China; 2 School of the First Clinical Medical Sciences, Wenzhou Medical University, Wenzhou, China; 3 Department of Gastroenterology, The Third Affiliated Hospital of Wenzhou Medical University, Wenzhou, China

## Abstract

**Purpose:**

We aimed to investigate the role of platelet-to-lymphocyte ratio (PLR) in cognitive decline in patients with type 2 diabetes mellitus (T2DM).

**Methods:**

A total number of 261 T2DM patients were enrolled in this study. The T2DM patients were divided into two groups according to the median of PLR (PLR < 96.5, *n* = 130; PLR ≥ 96.5, *n* = 131). Cognitive impairment was defined as Mini-mental State Examination score ≤ 26. Student’s *t* test and Chi-square test were used to test the difference between the groups, and logistics regression analysis were performed to verify whether high PLR was an independent factor for cognitive impairment.

**Results:**

T2DM patients with cognitive impairment had significantly higher PLR level when compared with the simple diabetes group (*p* = 0.003). Incidence of cognitive impairment was higher in the high PLR group, compared to low PLR group (*p* = 0.040). Multivariate logistic regression analysis suggested that PLR was a risk biomarker of cognitive decline in T2DM patients (odds ratio [OR] = 1.010, 95% CI: 1.001–1.018, *p* = 0.013).

**Conclusions:**

We demonstrated that a higher PLR was associated with cognitive decline in T2DM patients. The PLR may help to identify high-risk patients in time and provide clues for further prevention of cognitive dysfunction in T2DM patients.

## Introduction

With the accelerated pace of global population aging, type 2 diabetes mellitus (T2DM), one of the most common diseases among the elderly, has attracted more and more attention. Many studies have demonstrated that the negative effects of T2DM on cognitive function have significant clinical significance [1, 2]. In the course of diabetes, patients are more likely to progress to cognitive impairment and dementia, both can seriously affect the patients’ self-management, further exacerbating the disease and leading to more complications [1, 3]. Therefore, studies on risk factors for cognitive decline in T2DM patients have been carried out extensively. In the same way, we are eager to find a biomarker, which are accurate, reliable and easily accessible.

Clinical studies have shown that patients with T2DM are often accompanied by increased concentrations of various inflammatory factors and inflammatory markers that can predict the occurrence of T2DM, as well as induce or worsen diabetes. Inflammation has been reported to play a role in pathogenesis as one of the causes of cognitive dysfunction or dementia. The ratio of platelet-to-lymphocyte ratio (PLR) is a well-known marker of systemic inflammation [4, 5]. It has been studied as a biomarker of inflammation, showing great prognostic value as well as traditional markers of inflammation [6]. Therefore, in this study, we aimed to assess the relationship between PLR, which can be easily obtained from blood cell counts, and cognitive decline in patients with T2DM.

## Materials and methods

### Study population

From September to December 2018, we conducted a cross-sectional study of 300 patients with T2DM in the Third Affiliated Hospital of Wenzhou Medical University. The exclusion criteria were as follows: (1) hypoglycemic coma, diabetic ketoacidosis and other acute complications of diabetes (*n* = 2); (2) severe hepatic and renal insufficiency, severe systemic disease (Malignant tumor, thyroid disease, severe infection, severe anemia, etc.) (*n* = 11); (3) acute cardiovascular events, Parkinson’s disease, epilepsy, moderate depression or other mental disorders (*n* = 21); (4) severe loss of sight or hearing (*n* = 2); (5) missing data (*n* = 3). Finally, there were 261 patients left after excluding 39 patients who met the criteria. The research was approved by the Ethics Committee of the Third Affiliated Hospital of Wenzhou Medical University and obtained informed consent from all patients.

### Data collection

We collected demographic information of the patients through face-to-face questioning to gather information such as age, sex, years of education, smoking history, alcohol consumption history, diabetes course, etc. We performed a blood routine examination of the patients and obtained indicators like glycosylated hemoglobin (HbA1c), white blood cell (WBC), red blood cell (RBC), hemoglobin (Hb), triglyceride (TG), total cholesterol (TC), high density lipoprotein (HDL), low density lipoprotein (LDL), platelet (PLT), lymphocytes. WBC, RBC, Hb, HbA1c, PLT, lymphocytes were counted using XT-1800i (Sysmex, Kobe, Japan). TG, TC, HDL, LDL were counted using ARCHITECT c16000 (Abbott Laboratories, Illinois, USA). Platelet-to-lymphocyte ratio (PLR) was calculated as platelet/lymphocyte. What’s more, we assessed the cognitive status of all patients through Mini-mental State Examination (MMSE), which is one of the most widely used scales to assess whether people have cognitive impairment or not. In this study, diabetics were divided into two groups according to the MMSE score: the cognitive-unimpaired group (MMSE > 26, *n* = 190) and the cognitive-impaired group (MMSE ≤ 26, *n* = 71) [7, 8].

### Statistical analysis

All statistical analyses were performed in IBM SPSS Statistics 25.0 and all the figures were drawn by Graphpad Prism 7.0. The continuous variables of normal distribution were described by mean ± standard deviation and using Student’s *t* test to analyze the differences between groups. Continuous variables with non-normal distribution were represented by median and interquartile range of continuous variables, and the difference between groups was analyzed by Mann-Whitney U test. The categorical variables were described by counts or percentage, and Chi-square test was used for the comparison between groups. Univariate Logistics regression analysis was used to study the variables related to cognitive status of patients with T2DM. Multivariate logistic regression analysis was used to investigate the effect of PLR on the cognitive status of diabetic patients after controlling for confounding factors. *p* < 0.05 in all analyses was considered statistically significant.

## Result

### Baseline characteristics of the study subjects

**Fig. 1:**
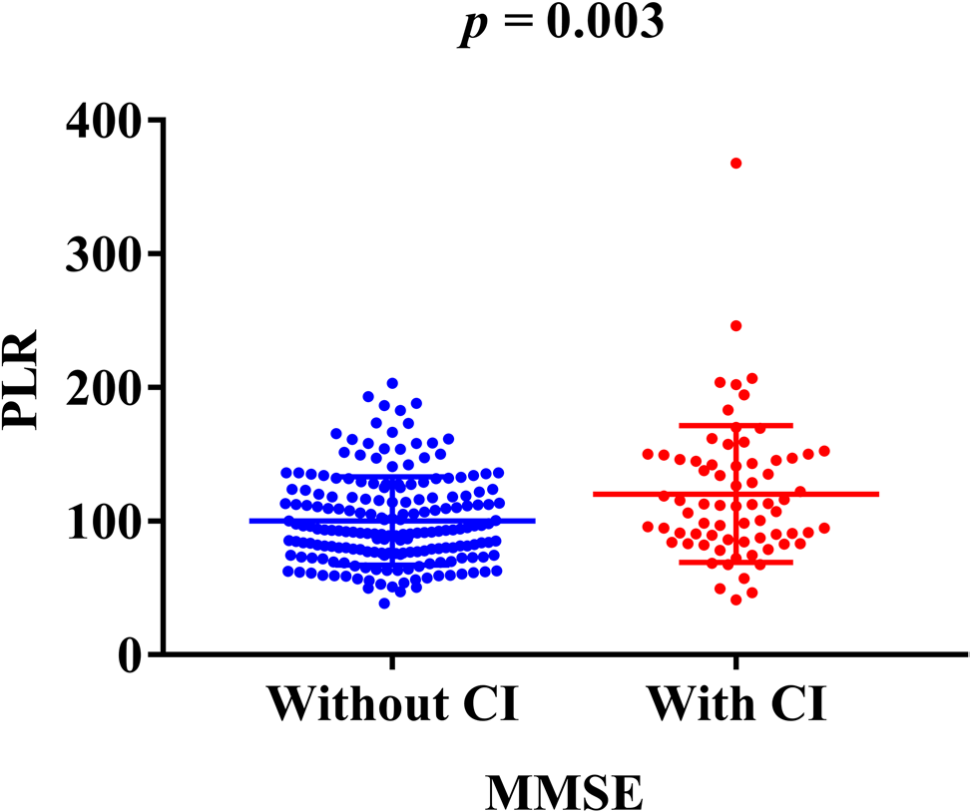
Comparisons of PLR in T2DM patients with cognitive impairment or not

Among the 261 diabetic patients, 190 were cognitive-unimpaired while 71 were cognitive-impaired. The baseline characteristics between groups with and without cognitive impairment were presented in Table 1. There were significant differences between the two groups in age (*p* < 0.001), sex (*p* < 0.001), education (*p* < 0.001), hypertension (*p* = 0.016), smoking (*p* < 0.001), drinking (*p* = 0.025), RBC (*p* = 0.001), Hb (*p* < 0.001), TC (*p* = 0.025) and PLR (*p* = 0.002). The cognitive impairment group was older, having higher proportion of women, prevalence of hypertension and higher level of PLR, but lower in the other respects than the non-cognitive impairment group. We can intuitively see that PLR in the cognitive impairment group was significantly higher than that in the non-cognitive impairment group (Fig. 1).

**Table 1:** Demographic and laboratory characteristics of simple diabetic patients and diabetic patients with cognitive impairment

Characteristics	Without CI (*n* = 190)	With CI (*n* = 71)	*p*
Age, (years)	55 (48–61)	60(56–67)	< 0.001
Sex, (male, n%)	141 (74.2)	31 (43.6)	< 0.001
BMI	24.0 (22.0–26.6)	24.0(22.5–26.7)	0.844
Education, (years)	7(4–9)	0 (0–2)	< 0.001
Diabetes duration, (years)	8 (3–11)	9 (5–16)	0.127
Hypertension, n (%)	73 (38.4)	39 (54.9)	0.016
Hyperlipidemia, n (%)	53 (27.8)	20 (28.1)	0.914
Smoking, n (%)	111 (58.4)	21 (29.5)	< 0.001
Drinking, n (%)	96 (50.5)	25 (35.2)	0.025
HbA1c	9.38 (7.96–11.28)	9.45 (7.83–11.33)	0.773
WBC, (×10^9^/L)	6.10 (5.10–7.20)	6.10 (5.30–7.40)	0.614
RBC, (×10^12^/L)	4.60 (4.32–4.90)	4.47 (4.15–4.71)	0.001
Hb, (g/L)	140.9 ± 14.73	55.02 (47.96–61.26)	< 0.001
TG, (mmol/L)	1.60 (1.06–2.35)	1.62(1.14–2.26)	0.675
TC, (mmol/L)	4.81 (3.86–5.52)	4.38 (3.69–5.01)	0.025
HDL, (mmol/L)	0.97 (0.84–1.18)	1.02 (0.85–1.16)	0.953
LDL, (mmol/L)	2.97 (2.22–3.58)	2.53(2.11–3.30)	0.073
PLR	92.94 (76.00–118.82)	111.82(84.62–146.11)	0.002

*CI* cognitive impairment, *BMI* body mass index, *HbA1c* glycosylated hemoglobin, *WBC* white blood cells, *RBC* red blood cells, *Hb* hemoglobin, *TG* triglyceride, *TC* total cholesterol, *HDL* high density lipoprotein, *LDL* low density lipoprotein, *PLR* platelet-to-lymphocyte ratio

### The association of PLR with cognitive function

Then all the diabetic patients were divided into G1 (PLR < 96.5, *n* = 130) and G2 (PLR ≥ 96.5, *n* = 131) groups according to the median of PLR. As shown in Table 2, diabetic duration (*p* = 0.002) and age (*p* = 0.034) in G2 group was significantly higher than that in G1 group. Apart from this, the figures of G2 group were lower than the G1 group in the other statistically significant indicators like sex (*p* < 0.001), education (*p* = 0.015), smoking (*p* < 0.001), drinking (*p* < 0.001), HbA1c (*p* = 0.036), RBC (*p* < 0.001), Hb (*p* < 0.001), TG (*p* = 0.001) and TC (*p* = 0.020).

**Table 2:** Characteristics of patients with diabetes mellitus with cognitive impairment according to PLR median

Characteristics	PLR < 96.5 (*n* = 130)	PLR ≥ 96.5 (*n* = 131)	*p*
With CI, n (%)	28 (21.5)	43 (32.8)	0.040
Age (years)	55 (49–61)	58 (52–64)	0.034
Sex (male, n%)	99 (76.1)	73 (55.7)	< 0.001
BMI	24.2 (22.3–26.8)	23.6 (21.9–26.0)	0.060
Education (years)	6 (2–9)	5 (0–8)	0.015
Diabetes duration (years)	6 (2–11)	9 (5–16)	0.002
Hypertension, n (%)	56 (43.0)	56 (42.7)	0.957
Hyperlipidemia, n (%)	35 (26.9)	38 (29.0)	0.736
Smoking, n (%)	83 (63.8)	49 (37.4)	< 0.001
Drinking, n (%)	75 (57.6)	46 (35.1)	< 0.001
HbA1c	9.71 (8.41–11.45)	9.27 (7.46–10.90)	0.036
WBC (×10^9^/L)	6.30 (5.50–7.30)	5.85 (5.00–7.20)	0.052
RBC (×10^12^/L)	4.71 (4.46–4.96)	4.41 (4.14–4.64)	< 0.001
Hb (g/L)	143.59 ± 14.58	133.07 ± 14.30	< 0.001
TG (mmol/L)	1.73 (1.25–2.76)	1.45 (0.96–2.14)	0.001
TC (mmol/L)	4.83 (4.05–5.56)	4.60 (3.72–5.31)	0.020
HDL (mmol/L)	0.96 (0.83–1.15)	1.02 (0.88–1.21)	0.167
LDL (mmol/L)	2.96 (2.24–3.61)	2.74 (2.08–3.50)	0.279

*CI* cognition impairment, *BMI* body mass index, *HbA1c* glycosylated hemoglobin, *WBC* white blood cells, *RBC* red blood cells, *Hb* hemoglobin, *TG* triglyceride, *TC* total cholesterol, *HDL* high density lipoprotein, *LDL* low density lipoprotein

### Increased PLR level is related to cognitive decline

**Fig. 2:**
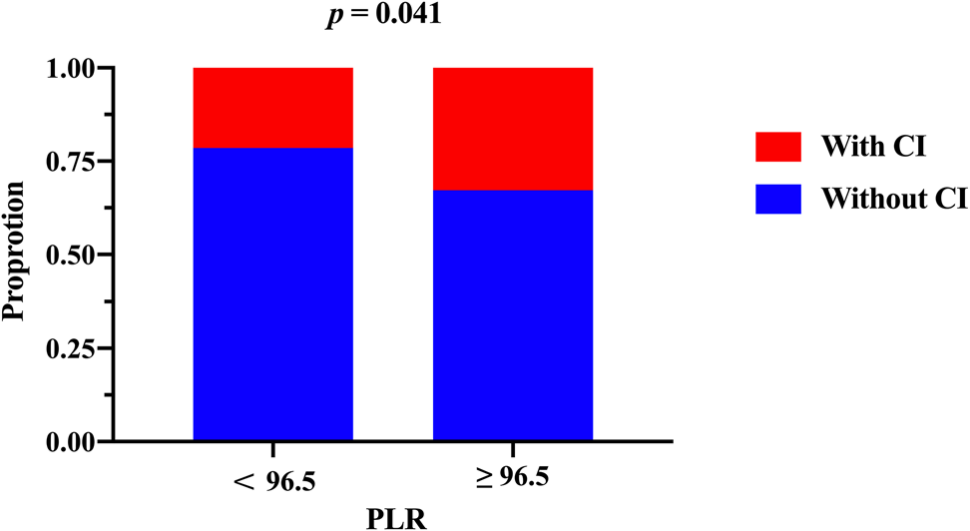
Comparison of the incidence of cognitive impairment of different PLR levels

**Fig. 3:**
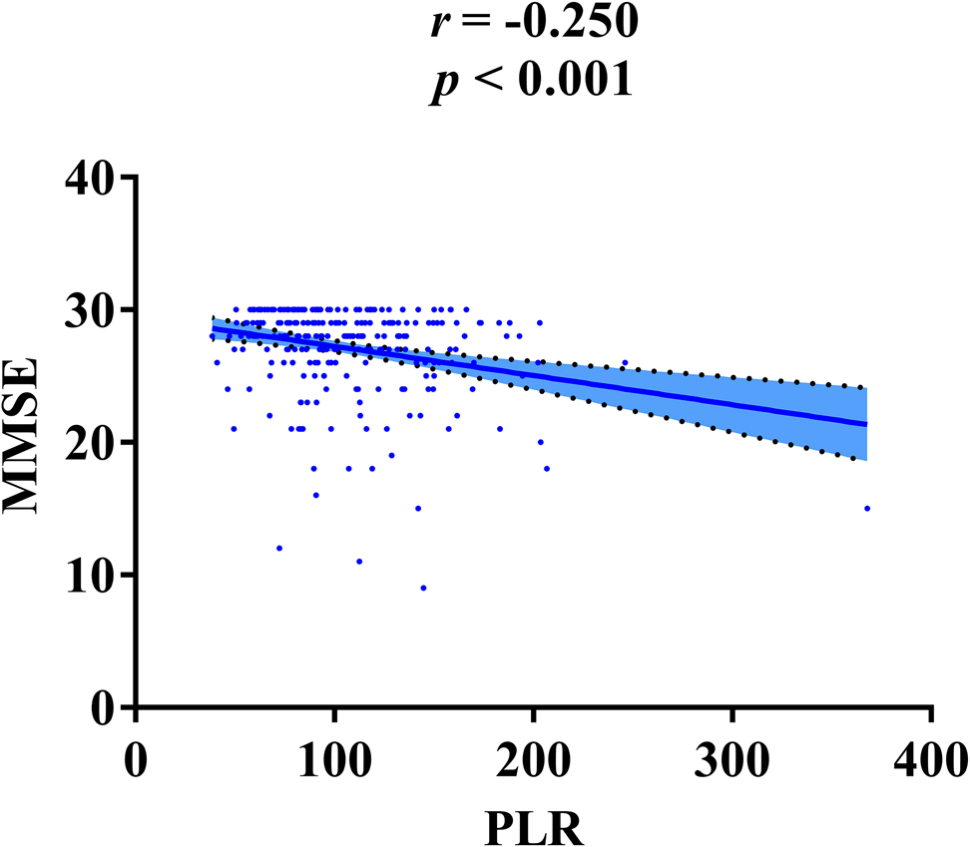
Correlation between PLR and MMSE. *r* = − 0.250, *p* < 0.001

In order to explore the distribution of patients with cognitive impairment in different concentration of PLR, we divided the patients into two groups through the median of PLR. 32.8% patients suffered from cognitive impairment in the group with a high PLR level (PLR ≥ 96.5) while only 21.5% patients suffered from cognitive impairment in the group with a low PLR level (*p =* 0.040) (Fig. 2). In addition, PLR and MMSE scores were found to be correlated through linear regression analysis (*r* = − 0.250, *p <* 0.001) (Fig. 3).

To further explore the relationship between PLR and cognitive impairment in diabetic patients, we performed a single-factor regression analysis. As shown in Table 3, age (*p* < 0.001), sex (*p* < 0.001), education (*p* < 0.001), hypertension (*p =* 0.017), smoking (*p* < 0.001), drinking (*p =* 0.026), RBC (*p =* 0.004), Hb (*p* < 0.001), PLR (*p =* 0.001) were significantly correlated with cognitive impairment in diabetic patients.

**Table 3:** Univariate logistic regression analyses for diabetes mellitus with cognitive impairment

Variables	Univariate logistic regression		
	OR	95% CI	*p*
Age, (years)	1.083	1.047–1.119	< 0.001
Sex, (male, n%)	3.713	2.098–6.570	< 0.001
BMI	0.976	0.894–1.065	0.582
Education, (years)	0.635	0.562–0.717	< 0.001
Diabetes duration, (years)	1.031	0.993–1.072	0.115
Hypertension, n (%)	0.512	0.295–0.889	0.017
Hyperlipidemia, n (%)	0.967	0.527–1.776	0.914
Smoking, n (%)	3.388	1.885–6.089	< 0.001
Drinking, n (%)	1.899	1.080–3.340	0.026
HbA1c	0.992	0.880–1.118	0.893
WBC (×10^9^/L)	1.040	0.896–1.207	0.607
RBC, (×10^12^/L)	0.428	0.241–0.760	0.004
Hb, (g/L)	0.958	0.939–0.977	< 0.001
TG, (mmol/L)	1.024	0.977–1.073	0.325
TC, (mmol/L)	1.003	0.882–1.139	0.969
HDL, (mmol/L)	0.944	0.382–2.330	0.900
LDL, (mmol/L)	0.768	0.565–1.044	0.092
PLR	1.013	1.005–1.020	0.001

*BMI* body mass index, *HbA1c* glycosylated hemoglobin, *WBC* white blood cells, *RBC* red blood cells, *Hb* hemoglobin, *TG* triglyceride, *TC* total cholesterol, *HDL* high density lipoprotein, *LDL* low density lipoprotein, *PLR* platelet-to-lymphocyte ratio

Multivariate logistic regression analyses were performed to control other potential confounding variables (Table 4). In Model 1, nothing was adjusted (odds ratio [OR] = 1.013, 95% CI:1.005–1.020, *p* = 0.001). After adjusting for age, sex in Model 2, the linkage between PLR and cognitive impairment remained significant (odds ratio [OR] = 1.010, 95% CI: 1.002–1.018, *p* = 0.014). On the basis of Model 2, we additionally made adjustments for smoking and drinking in Model 3, the linkage between PLR and cognitive impairment still remained significant (odds ratio [OR] = 1.010, 95% CI: 1.001–1.018, *p* = 0.013). After two adjustments, there was still a correlation between PLR and cognitive impairment, which showed that PLR was probably an independent impact factor of T2DM patients with cognitive impairment.

**Table 4:** Adjusted Odds Ratio (95% Confidence Interval) for T2DM with cognitive impairment

Variables	OR	95% CI	*p*
Model 1	1.013	1.005–1.020	0.001
Model 2	1.010	1.002–1.018	0.014
Model 3	1.010	1.002–1.018	0.013

Model 1 is univariate analysis

Model 2 is adjusted by age and sex

Model 3 is adjusted by age, sex, smoking and drinking

## Discussion

In this cross-sectional study, we investigated the relationship between PLR and cognitive decline in T2DM in 261 patients from the Third Affiliated Hospital of Wenzhou Medical University. The main findings of this study were as follows: (1) PLR was significantly correlated with cognitive function in patients with type 2 diabetes; (2) After adjusting for age, sex, smoking and alcohol consumption, the *p* value of PLR was still significant. Our study found that PLR levels in T2DM patients combined with cognitive impairment were higher than those in simple T2DM patients.

As far as we know, T2DM is a chronic metabolic disease caused by insulin resistance and insufficient insulin secretion compensation response. Inflammatory pathways are considered as potential agents of diabetes [9]. The mechanisms that trigger inflammation in T2DM remain unclearly. Inflammation may promote the development of T2DM by causing insulin resistance, while hyperglycemia may exacerbate the inflammatory response, thus promoting the long-term complications of diabetes [10]. In the course of inflammatory response, the continuous production of pro-inflammatory factors such as interleukin-6 (IL-6), interleukin-1β (IL-1β) and tumor necrosis factor-α (TNF-α) [11, 12] can cause various nervous system lesions, including amyloidosis, neuronal death [13, 14], cortical thinning [15, 16], reduced brain volume [15], cerebral vascular disease related events such as micro hemorrhage, infarcts [17, 18] and neurodegeneration [2]. These lesions can lead to cognitive decline.

PLR, which represents the balance between platelet and lymphocyte levels, having been recognized as an indicator of inflammatory status in patients with a variety of chronic inflammatory diseases [19]. Baodong Qin et al. [20] showed that PLR level was significantly increased in patients with rheumatic diseases compared with normal subjects. Meanwhile, Guang Shi et al. [21] reported that PLR of asthmatic critically ill patients was significantly higher than that of non-critically ill patients and control group. Apart from this, van der Willik et al. [22] found that PLR levels were associated with lower cognitive performance in the study of breast cancer survivors. In our study, PLR of patients with cognitive impairment was significantly higher than that of patients with type 2 diabetes alone. Studies have found that high levels of PLR are associated with activity and poor prognosis in a variety of diseases, including chronic atrophic gastritis [23], colorectal cancer [24] and osteosarcoma [25]. PLR may be more reliable as a combination of these two markers due to the complex interaction between platelets and lymphocytes. And it is a very easy indicator to be calculated.

Our study has some certain limitations. Firstly, this is a single-center cross-sectional study, with the possibility of selection bias, and the generalization of the findings to clinical fields should be cautious. Secondly, the sample size is relatively small. In addition, the MMSE score alone may not be comprehensive enough to assess patients’ cognitive status. Thirdly, certain drugs have effects on cognitive impairment, but this was not included in our data analysis. Finally, we only discussed the clinical significance of PLR and cognitive decline in T2DM patients, lacking the research on the specific mechanism. Therefore, in future studies, prospective cohort studies should be designed to expand the scope and number of sample collection, combined with more indicators to evaluate cognitive function, and further study the mechanism of PLR in the process of cognitive decline in T2DM patients, so as to provide greater referential significance for clinical work.

## Conclusions

We demonstrated that a higher PLR level was associated with cognitive decline in T2DM patients. The PLR may help to identify high-risk patients in time and provide clues for further prevention of cognitive dysfunction in T2DM patients.

## Data Availability

Data included in the current study are not publicly available to ensure confidentiality of the patients but are available from the corresponding author on reasonable request.
